# Towards a Rational Design of a Continuous-Flow Method for the Acetalization of Crude Glycerol: Scope and Limitations of Commercial Amberlyst 36 and AlF_3_·3H_2_O as Model Catalysts

**DOI:** 10.3390/molecules21050657

**Published:** 2016-05-18

**Authors:** Sandro Guidi, Marco Noè, Pietro Riello, Alvise Perosa, Maurizio Selva

**Affiliations:** Department of Molecular Sciences and Nanosystems, Centre for Sustainable Technologies, Ca’ Foscari University of Venice, Via Torino 155, 30175 Venezia Mestre, Italy; sandro.guidi@unive.it (S.G.); marco.noe@unive.it (M.N.); riellop@unive.it (P.R.); Alvise@unive.it (A.P.)

**Keywords:** acetalization, glycerol, solketal, catalysis, continuous-flow

## Abstract

The acetalization of six different types of glycerol including pure, wet, and crude-like grade compounds of compositions simulating those of crude glycerols produced by the biodiesel manufacture, was carried out with two model ketones such as acetone and 2-butanone. The reaction was investigated under continuous-flow (CF) conditions through a comparative analysis of an already known acetalization catalyst such as Amberlyst 36 (A36), and aluminum fluoride three hydrate (AlF_3_·3H_2_O, AF) whose use was never previously reported for the synthesis of acetals. At 10 bar and 25 °C, A36 was a highly active catalyst allowing good-to-excellent conversion (85%–97%) and selectivity (99%) when either pure or wet glycerol was used as a reagent. This catalyst however, proved unsuitable for the CF acetalization of crude-like glycerol (CG) since it severely and irreversibly deactivated in a few hours by the presence of low amounts of NaCl (2.5 wt %) which is a typical inorganic impurity of raw glycerol from the biorefinery. Higher temperature and pressure (up to 100 °C and 30 bar) were not successful to improve the outcome. By contrast, at 10 bar and 100 °C, AF catalyzed the acetalization of CG with both acetone and 2-butanone, yielding stable conversion and productivity up to 78% and 5.6 h^−1^, respectively. A XRD analysis of fresh and used catalysts proved that the active phase was a solid solution (SS) of formula Al_2_[F_1-x_(OH)_x_]_6_(H_2_O)_y_ present as a component of the investigated commercial AF sample. A hypothesis to explain the role of such SS phase was then formulated based on the Brønsted acidity of OH groups of the solid framework. Overall, the AF catalyst allowed not only a straightforward upgrading of CG to acetals, but also a more cost-efficient protocol avoiding the expensive refining of raw glycerol itself.

## 1. Introduction

**Scheme 1:**
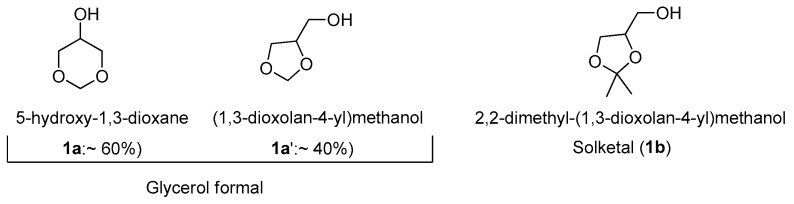
Most common cyclic acetals derived from glycerol. Glycerol formal is a 3:2 mixture of six- and five-membered ring isomers.

The synthesis of cyclic acetals of glycerol (GAs), particularly glycerol formal and solketal, is among the promising routes for the chemical exploitation of the glut of glycerol originating from biodiesel production (Scheme 1) [1,2,3].

Both glycerol formal and solketal, and more generally, cyclic acetals obtained by the condensation of glycerol with light (up to C_4_) aldehydes and ketones, display an array of intriguing properties. Like glycerol, they are viscous, dense, non-toxic, and thermally stable liquids with boiling points in the proximity and over 200 °C [4,5]. However, since they derive from the formal protection of two OH groups of glycerol, they possess polarity, hydrophobicity, and hydrogen bonding ability that make them similar to simple aliphatic alcohols [6]. These aspects account for major applications of GAs as safe solvents in the formulation of injectable preparations, paints, plastifying agents, insecticide delivery systems, and flavors [1,6,7].

Moreover, the stability of glycerol acetals to oxidative conditions and their miscibility with biodiesel blends, have been key features to investigate their potential as renewable ashless fuel additives. Open and patent literature reports demonstrate that the incorporation of GAs in fuels can improve the quality of both standard (petrochemical-based) and bio-diesels by reducing particulate emissions, viscosity, and pour point [1,8,9,10,11].

Last, but not least, GAs possess a short OH-capped tether (hydroxymethylene group) which provides synthetic access to a number of other derivatives, mainly ethers, esters, and carbonates [2,8,12,13].

The broad spectrum of applications and interests for cyclic acetals of glycerol has also triggered research towards improving the performance of acetalization catalysts. Acetalization reactions are conventionally carried out with strong mineral acid catalysts such as sulfuric, hydrohalic, and *p*-toluenesulfonic [14,15,16]. Although these are all quite active systems, a drawback of such processes, especially for the synthesis of GAs, is the formation of water as an equilibrium by-product: this not only weakens the acid strength and decreases the conversion of glycerol, but also entails corrosion and work-up problems [17]. Azeotropic removal of water with hydrocarbons or halogenated solvents offers an expedient to cope with this difficulty [18], though it is rather uneconomic and dangerous for large scale preparations, and it becomes impracticable when low boiling carbonyl reactants (e.g., acetone) are used [15,19]. Far more elegant and eco-friendly solutions can be implemented through several types of catalytic systems. Among these: inorganic Fe- and B-based salts able to act as both Lewis acid catalysts and dehydrating agents [20], and solid acids including zeolites, heteropolyacids, modified meso-structured silicas, and transition metal oxides/complexes have been reported [7,19,21,22]. Brønsted acidic ionic liquids (e.g., *N*-butyl- pyridinium bisulfate, [BPy][HSO_4_]) have also been recently introduced as water-removal micro catalytic reactors for the synthesis of GAs [23].

As a part of our research is interested in the chemical valorization of biomass derivatives [12,13,24,25], we also focused on the glycerol acetalization aimed at achieving a robust catalytic method operating under continuous-flow (CF) conditions and possibly, suitable to the conversion of crude glycerol (CG) as obtained from biodiesel plants. In fact, the upgrading of CG offers great opportunities not only to promote the commercialization of glycerol derivatives, but also to defray costs of biofuels [26].

To the best of our knowledge, the CF-preparation of GAs was reported in very few papers: [27,28,29,30,31] these proved the efficiency of acid catalysts such as Amberlyst (15 and 36) resins and sulfuric acid, but also highlighted clogging issues and deterioration/deactivation of the reactors and catalytic beds, due to the viscosity of reactants and products, and to the co-formation of water. These problems pushed to engineering improvements of the CF-technologies through the use of semi-batch or corrosion-resistant glass reactors, co-solvents, and even subcritical reagents.

In this context, we considered commercially available aluminum fluoride trihydrate (AlF_3_·3H_2_O, AF), as a potential new catalyst for the formation of GAs in CF-mode. AF as such, or its dehydrated-, partly hydroxylated-, nano- and supported-forms have been proposed as acid catalysts for several processes such as halide exchanges on hydrochlorocarbons [32,33], aromatic alkylations [34,35], hydrocarbon isomerizations [36], and condensation processes [37]. Moreover, AF is a safe [32,33,34,35,36,37,38], highly thermally (up to 400 °C) and mechanically stable, and relatively inexpensive compound.

The present work compares the unprecedented use of AlF_3_·3H_2_O as an acetalization catalyst to that of a model acid solid such as Amberlyst 36. In particular, the analysis of the CF-reaction of glycerol with acetone demonstrates that the sulfonated resin (Amberlyst) is definitely more active than AF when pure or even wet reactants are used. AF however, is a far superior catalyst for the transformation of crude glycerol: for example, at 100 °C and 10 bar, mixtures of glycerol, methanol, water, and NaCl (with compositions mimicking those of off-grade glycerol produced in the biodiesel manufacture) readily reacts with acetone to give the corresponding acetal (solketal) very good conversion and selectivity (≥80% and 99%, respectively). Under such conditions, the catalytic bed of AF has been operated without any drop of performance, clogging, or appreciable catalyst leaching. By contrast, the Amberlyst resin is completely and irreversibly deactivated when used with raw glycerol, particularly by NaCl present as an impurity in the reactant stream.

A XRD analysis has allowed to identify an aluminum hydroxide fluoride solid solution of formula A_l_[F_1-x_(OH)_x_]_3_(H_2_O)_y_ as the phase responsible for the activity of investigated AF catalyst.

The CF-acetalization protocol has been successfully extended to the reaction of glycerol with 2-butanone catalyzed by AF. Though, the lower solubility of such a ketone (with respect to acetone) in both pure and crude-like glycerols makes the process less tolerant to water and other impurities in the reactants stream.

Overall, the design of the investigated CF-reaction has been rationalized by considering the nature, the grade, and the reactivity of the starting glycerol and the carbonyl compounds used.

## 2. Results

The experimental apparatus used for the investigation of CF-acetalyzation reactions was similar to that described in our previous papers: [13,39] it was composed of an HPLC pump for the delivery of liquid reactants (glycerol and the selected ketone), a thermostated oven equipped with additional thermocouples for temperature control, a static mixer and a stainless steel tubular reactor (placed in the oven) and a back-pressure regulator (BPR) for pressure control. All reagents and the catalyst were ACS grade and used as they were supplied from Sigma-Aldrich (Milan, Italy. See experimental for further details).

### 2.1. The CF-Acetalization of Glycerol with Acetone

The model acetalization of glycerol with acetone was initially investigated in the presence of the most active catalysts previously reported for the process, Amberlyst 36 (A36), in order to set up the CF-system. AlF_3_·3H_2_O was then considered to verify whether it could act as a reaction catalyst, and in that case, to compare its performance to that of A36. A wide range of conditions were explored starting from tests on different grades of glycerol: pure and blended with methanol, variable amounts of water, and NaCl. The relative proportions of each additive were chosen not only to operate with homogeneous solutions, but also to simulate the composition of crude glycerol (CG) deriving from biodiesel processes [26,40,41,42]. In fact, typical CG composition is <65 wt % glycerol, 15–50 wt % MeOH, 10–30 wt % water, and 2%–7% salts (primarily NaCl and KCl). Table 1 summarizes the types of glycerol used in this investigation.

Due to solubility limitations, the use of pure glycerol (**Glyc1**) required a 40 molar excess of acetone to achieve a homogeneous solution of reactants. For **Glyc2–5** and **Glyc6**, the acetone:glycerol molar ratio (Q) was set to 4 and 8, respectively. 

Other conditions were adjusted according to those described in previous papers: [29,30,31] tests were carried out at temperature and pressure from 25 to 100 °C, and 2 to 35 bar [43], respectively. Screening experiments allowed to choose the size of the reactor, the catalyst loading, and the weight hourly space velocity (WHSV, [44]). These indicated that reactions could be conveniently carried out using a cylindrical steel reactor (V = 0.875 mL; L = 12 cm; Ø = 1/4″) filled with the catalyst (Amberlyst 36: 0.90 g; AlF_3_·3H_2_O, 0.67 g), and fed in the upright position with the reactant mixture at a WHSV of 2 (g_glycerol_·h^−1^·g_cat_^−1^). Since the acid loading of A36 was 5.1 meq/g (from Sigma-Aldrich), AF was used in a molar amount comparable to the acid equivalents available in the resin. All experiments were monitored for 24 h by sampling the reaction mixture periodically and analysis by GC and GC/MS in order to evaluate both the glycerol conversion and the product distribution.

**Scheme 2:**
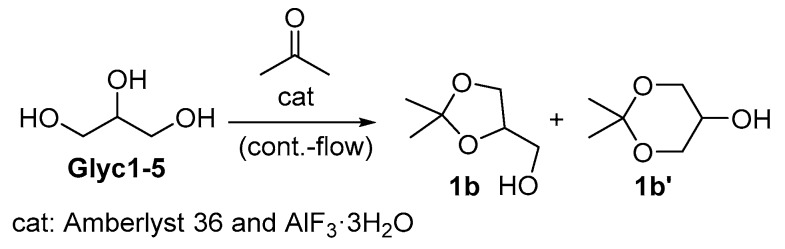
The CF-acetalization of glycerol with acetone.

**Figure 1:**
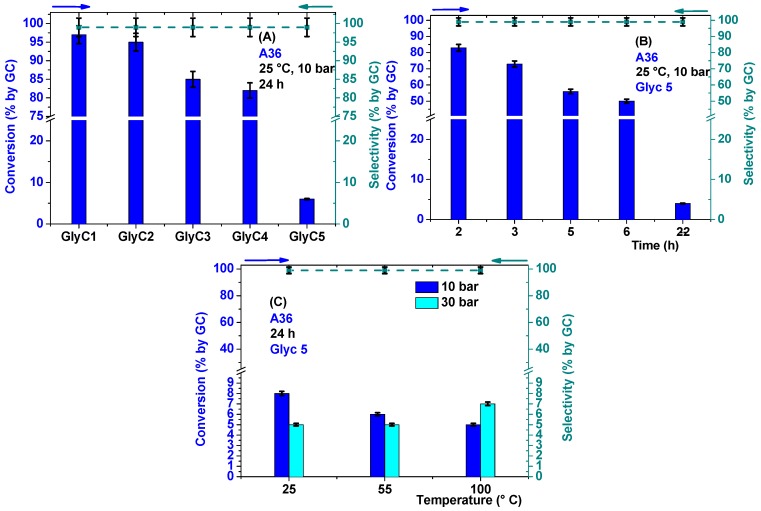
CF-reaction of glycerol with acetone over Amberlyst 36. (**A**) Conversion of glycerol achieved after 24 h, at 25 °C and 10 bar, by feeding different types of reactant **Glyc1-5** to the reactor; (**B**) Profile of the glycerol conversion *vs.* time obtained at 25 °C and 10 bar with the use of **Glyc5** as the reagent; (**C**) Effect of T and p on the conversion of **Glyc5** achieved after 24 h. In all cases, WHSV = 2 was used. The molar ratio acetone:glycerol was 40 and 4 for **Glyc1** and **Glyc2-5**, respectively.

**Figure 2:**
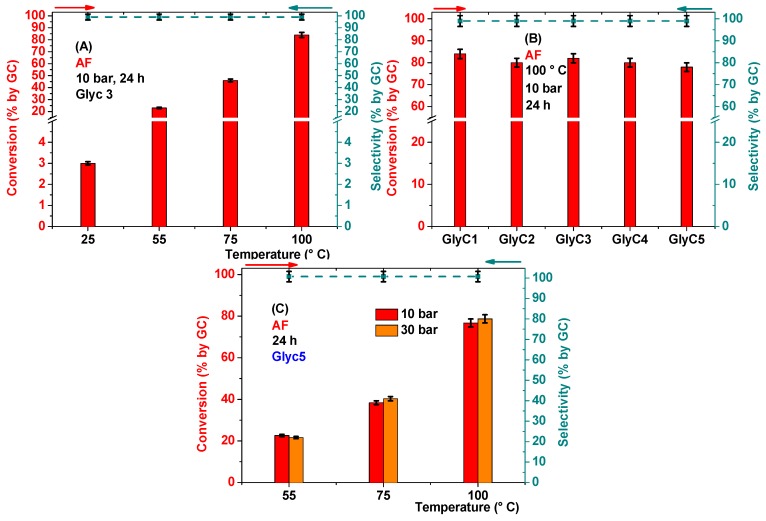
CF-reaction of glycerol with acetone over AlF_3_·3H_2_O. (**A**) Profile of the glycerol conversion *vs.* temperature obtained at 10 bar, after 24 h, with the use of **Glyc3** as the reagent; (**B**) Conversion of glycerol achieved after 24 h, at 100 °C and 10 bar, by feeding different types of reactant **Glyc1-5** to the reactor; (**C**) Effect of T and p on the conversion of **Glyc5** achieved after 24 h. In all cases, WHSV = 2 was used. The molar ratio acetone:glycerol was 40 and 4 for **Glyc1** and **Glyc2-5**, respectively.

Figure 1A–C and Figure 2A–C report the result obtained for the CF-acetalization of **Glyc1–5** with acetone over Amberlyst 36 and AlF_3_·3H_2_O, respectively (Scheme 2). Except for Figure 1B, conversions and selectivity were determined after 24 h. Each test was triplicated to check for reproducibility [45].

Amberlyst 36 was a highly efficient catalyst: at 25 °C, we noticed that it (A36) was active also operating at a lower pressure (10 bar) than that previously reported (30 bar, [29,30,31]). Under such conditions, a substantially quantitative process was observed when either pure glycerol or an almost equimolar glycerol/MeOH mixture were used (Figure 1A: **Glyc1** and **Glyc2**, respectively). A still satisfactory conversion of 82%–85% was reached even in the presence of sizeable amounts of water (from 4 up to 24 wt %) in the reactant stream (**Glyc3** and **Glyc4**, respectively). The overall selectivity–defined as the percentage ratio of the desired acetalization product (total of isomers **1b** and **1b′**) with respect to the conversion–was always >99%: isomeric acetals **1b** (solketal, preferred) and **1b′** were obtained in an approximately constant relative ratio of ~50 (dashed green profiles of Figure 1A–C).

The structures of products (**1b** and **1b′**) were assigned by GC/MS analyses and by comparison to an authentic commercial sample of solketal (**1b**). GC and GC/MS records also indicated that both the conversion and the product distribution did not undergo any appreciable change from the first two hours up to the end (24 h) of each CF-reaction. Moreover, additional experiments—not shown here—with both **Glyc2** and **Glyc4** proved that the catalytic bed could be reused for at least 48 h without loss of activity or selectivity.

However, the catalytic performance of A36 was dramatically affected by the addition of NaCl which is one of the main impurities of crude glycerol coming from the saponification and acidification steps involved in biodiesel production [23]. The CF-acetalization of **Glyc5** that contained only 2.5 wt % of NaCl, showed a progressive drop of the reaction conversion from 82% to 4% in 22 h (Figure 1B). After that time, the catalyst was no longer effective. Any attempt to improve such an outcome by using a fresh catalytic bed of A36 at higher temperature and pressure proved unsuccessful (Figure 1C): even at 100 °C and 30 bar, the glycerol conversion never exceeded 7% and it was comparable to that observed during blank runs (not shown here) carried out in the absence of any catalyst. As reported also by other authors [19,46], the reaction could not be promoted thermally. On the other hand, reported procedures for the reactivation the Amberlyst resin by mineral acids (e.g., H_2_SO_4_) were not only time-consuming and corrosive, but also ineffective in restoring the initial performance of the catalyst [29].

AlF_3_·3H_2_O was then tested. Commercial AlF_3_·3H_2_O (from Sigma-Aldrich) was used as such and after calcination in air at 500 °C for 5 h carried out according to an already reported procedure: [47] the two samples were labelled as AF and AF_c_, respectively. Although AF had never been previously reported as an acetalization catalyst, experiments demonstrated that in its presence, the investigated reaction was feasible. By contrast, the calcined compound AF_c_ proved totally inefficient. Two major facts emerged by the comparison of AF to A36: (i) AF was less active than the Amberlyst resin when both pure and wet glycerol (**Glyc1–4**) were used. Consider for example, the model acetalization of **Glyc3** with acetone. Under the same set of conditions (25 °C, 10 bar, WHSV = 2, molar ratio acetone:glycerol = 4, 24 h), the glycerol conversion was 3% and 85% over AF and A36, respectively (compare Figure 1A and Figure 2A). Only by rising the temperature up to 100 °C, the reaction proceeded further to reach a steady 84% conversion also over the AF catalyst (Figure 2A). A further experiment carried out by using the same catalytic bed for additional 30 h, proved that both conversion (of **Glyc3**) and selectivity did not change over time (83 and >99%, respectively), thereby confirming the robustness of the AF system; (ii) Notwithstanding the demand for a higher reaction temperature (100 °C), AlF_3_·3H_2_O was able to induce not only the acetalization of **Glyc1–4**, but also that of **Glyc5**: in all cases, the glycerol conversion was in the range of 78%–84% (Figure 2B) with >99% selectivity towards products **1b** and **1b′** (dashed green profile). Regardless of conditions and composition of the reactant mixture, the **1b**/**1b′** ratio was rather constant (~40) and comparable to that achieved with A36. Such results were confirmed by both prolonging the reaction of **Glyc5** up to a time-on-stream of 48 h and by reusing the same catalytic bed for three subsequent tests under the conditions of Figure 2B. This proved that AF allowed a stable and reproducible protocol, and it was a far superior catalyst than A36 for the transformation of wet glycerol contaminated by NaCl (**Glyc5**).

Mixtures recovered after the acetalization of **Glyc3** and **Glyc4** over AF were subjected to ICP and ionic chromatography analyses for the determination of Al and fluoride contents. The Al and fluoride concentrations were found to be ~200 ppb and 1.91 ppm, respectively, corresponding to a mass loss of the catalytic bed of maximum <0.6 mg per 40 working hours (8 h/day per one week), with an insignificant incidence on the overall process [48].

Additional experiments also demonstrated that in the presence of AF catalyst, the reaction of **Glyc5** with acetone was improved by an increase of the temperature in the same way described for **Glyc3** (compare Figure 2A,C in the interval between 55 and 100 °C); though, the conversion profiles did not change when the pressure was raised from 10 to 30 bar (Figure 2C), this effect resembling that shown in Figure 1C.

Overall, the use of AlF_3_·3H_2_O allowed unprecedented good results, otherwise not possible with the Amberlyst resin, for the reaction of a crude-like glycerol such as **Glyc5** with acetone to produce the corresponding acetal (solketal) via a straightforward CF-mode.

The study was then continued to further explore the potential of AF as an acetalization catalyst.

### 2.2. AlF_3_·3H_2_O for the Acetalization of **Glyc5** and **Glyc6**: Reaction Productivity

The reaction of the two crude-like glycerols **Glyc5** and **Glyc6** was compared by using different acetone:glycerol (Q) molar ratios, WHSVs, and catalyst loadings. In order to increase the Q ratio, the content of MeOH and water of **Glyc6** were adjusted to both lower and higher values, respectively, with respect to **Glyc5** (Table 1): this choice allowed us to overcome limitations of mutual solubility of reactants and to operate with up to 8 molar equivalents. excess of acetone (Q = 8) with respect to glycerol. Experiments were all carried out at 100 °C, 10 bar, and for 24 h. The total volumetric flow rate (F) was in the range of 0.1 to 1.18 mL/min, and the corresponding WHSV was from 2 to 6. Also, two catalyst loadings of 0.67 g (same as for Figure 2) and 4.2 g were considered. Each test was triplicated to check for reproducibility. (See note 45) Table 2 report the results. For a more convenient comparison, the reaction productivity (P), expressed as the mass of product (total of isomers **1b** and **1b′**) obtained per hour and per mass unity of the catalyst (g of (**1b** + **1b′**)/(g_cat_ h)) is indicated.

The reaction of **Glyc5** showed that by keeping all the other conditions unaltered with respect to Figure 2B,C, an almost linear rise of the productivity from 2.2 to 5.6 h^−1^ was observed when the WHSV was tripled (entries 1–3). Although no other increments of WHSV were tested, it was plausible that the catalytic bed was still not operating at its maximum capacity. The result led to the conclusion that the process could be efficiently intensified and the yield of solketal could be further improved. However, at a constant WHSV of 2, the productivity remained steady at 2.2 h^−1^ even if the catalyst loading was increased six-fold (from 0.67 to 4.2 g; compare entries 1 and 4). This proving that the reaction reached an equilibrium conversion not exceeding 78%.

A similar behavior was observed for the acetalization of **Glyc6**, though with a lower productivity: under the same conditions, P of 2.2 and 0.86 h^−1^ were obtained for **Glyc5** and **Glyc6**, respectively (compare entries 1 and 5). Only by doubling the amount of acetone (Q = 8), P was improved up to values comparable to those achieved for **Glyc5** (compare entries 1–2 to 6–7). Also in this case however, operating at a constant WHSV of 2, the increase of the catalyst loading had no substantial effects on the rate of formation of the product (entries 6 and 8: *p* = 1.7 and 1.6 h^−1^, respectively). The poorer performance of the catalyst observed with the use of **Glyc6** was plausibly due to the higher water content (49 wt %) of this reagent with respect to **Glyc5** (24 wt %). Notwithstanding this, the catalytic bed proved robust and able to offer stable and reasonably good conversions over time.

Data of Table 2 were validated by the mass balance: as an example, after the reaction of entry 3, the vacuum distillation of the mixture allowed to isolate the product (as a mixture of isomer acetals **1b** and **1b′**) in a 59% yield. This compound as such was of ACS grade (>99%) and no additional purification steps were required.

### 2.3. The CF-Acetalization of Glycerol with 2-Butanone

The acetalization of glycerol with 2-butanone was examined to extend the synthetic scope of the investigated protocol. Experiments were carried out according to conditions of Figure 2B and Table 2, by using **Glyc1** and **Glyc6** as reagents. Since the solubility of pure glycerol (**Glyc1**) in 2-butanone was lower than that in acetone, the reactant molar ratio (Q, **Glyc1**:2-butanone) was set to 60 to achieve a homogeneous mixture upstream of the catalytic bed. For the same reason, the reaction of **Glyc6** required additional MeOH (0.5 extra mL of MeOH/mL **Glyc6**) as a co-solvent. Experiments were carried for 24 h at 100 °C, 10 bar, and WHSV of 2 h^−1^. Each test was triplicated to check for reproducibility. (See note 45) Results are reported in Table 3.

**Scheme 3:**
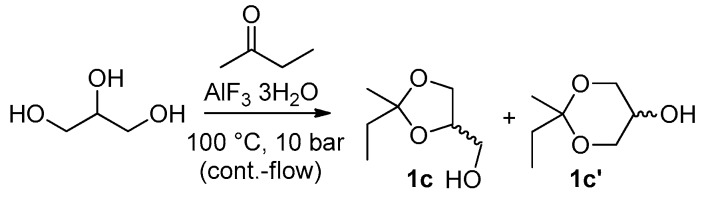
The reaction of glycerol with 2-butanone.

A steady conversion of 85% and 45% was achieved for the acetalization of **Glyc1** and **Glyc6**, respectively, and the corresponding productivity (P) was 2.7 and 1.3 g(**1c** + **1c′**)/(g_cat_ h) on the total formation of isomer products **1c** and **1c′** (Scheme 3).

The process was not further optimized, but the results proved the concept: AF was an efficient catalyst also for the reaction of other carbonyl compounds with crude-like glycerol. The productivity with 2-butanone was apparently lower than that with acetone; however, a direct comparison between the reactivity of two ketones could not be inferred since different reactant molar ratios as well as amounts of MeOH must be used in the tests of Figure 2, Table 2 and Table 3.

**Scheme 4:**
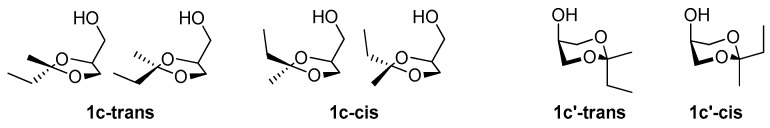
Conformational isomers for the reaction of glycerol with 2-butanone.

As far as the product distribution, the reaction of 2-butanone could, in principle, afford the following isomer products (Scheme 4).

In the five-membered ring compound **1c**, the C2 and C4 asymmetric carbons of the dioxolane ring could give rise to a DL-isomers pair, while two *cis-trans* isomers of acetal **1c’** were possible due to the relative axial and equatorial positions of ethyl and methyl substituents at the C5 of the dioxane ring. Accordingly, experiments of Table 3 showed the formation of almost equimolar amounts of **1c-*cis*** and **1c-*trans*** as major products along with two other minor compounds which were plausibly the *cis* and *trans* isomers **1c’**. Derivatives **1c** (*cis + trans*) could not be separated from each other, but their (1:1) mixture was isolated and characterized by NMR and MS. On the contrary, any attempt to obtain isomers **1c’** (*cis + trans*, either separately or in mixture) failed because they formed in low amounts. The structure of such compounds was hypothesized from GC/MS analyses of final reaction mixtures [49]. (Further details are reported in the experimental and Supplementary Materials sections).

### 2.4. Characterization of AlF_3_·3H_2_O

**Figure 3:**
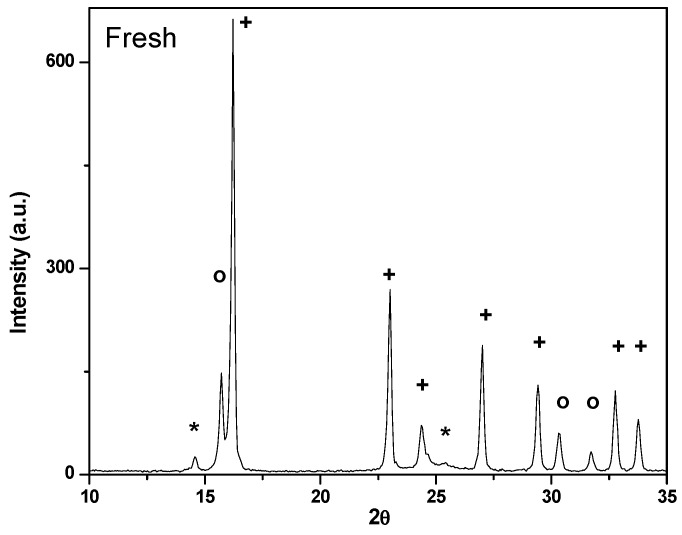
Diffraction pattern of the AF sample: (**+**) AlF_3_ (H_2_O)_3_ ICSD 416689 (83 wt %); (**o**) Al_2_ [(O H)_x_ F_(1-x)_]_6_ (H_2_O)_y_ COD 1000086 (12.9 wt %) and (*) β-AlF_3_ ICSD 202681 (3.2 wt %).

**Figure 4:**
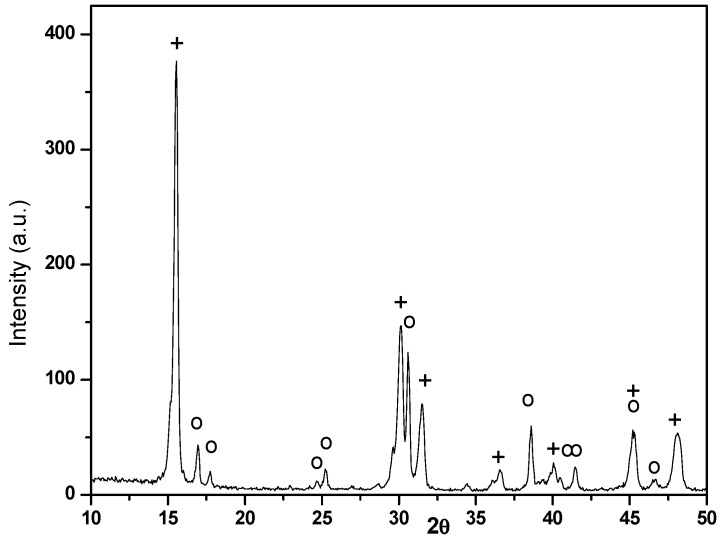
Diffraction pattern of AF_2_ sample: (**+**) Al_2_[(OH)_x_F_(1-x)_]_6_(H_2_O)_y_ COD 1000086 (63.4 wt %); (**o**) Al_3_F_14_Na_5_ ICSD 26419 (36.6 wt %).

A total of four different samples of fresh, calcined, and used AlF_3_·3H_2_O were considered for XRD characterization analyses (Table 4). Results are reported in Figure 3 and Figure 4.

X-ray powder diffraction (XRPD) of the fresh commercial catalyst AF sample showed that it was comprised of three phases: AlF_3_·3H_2_O, Al_2_[(OH)_x_F_(1-x)_]_6_(H_2_O)_y_, and β-AlF_3_ which were in the relative weight amount of 83%, 12.9%, and 4.1%, respectively. (Figure 3; Rietveld analysis of the XRD data was used to obtain the quantitative fractions of all phases. See Supplementary Materials for further details. Figure S6).

The most abundant phase was the expected AlF_3_·3H_2_O, while the second major component was identified as a solid solution (SS) of AlF_3_ and Al(OH)_3_ having the formula Al_2_[(F_1-x_(OH)_x_]_6_(H_2_O)_y_. This crystalline aluminum hydroxide fluoride (SS) showed a cubic pyrochlore structure cell with 16 formula units where aluminum atoms were centered in corner-shared AlF_x_O_6-x_ octahedrons forming a network of channels. F and OH groups were statistically distributed at the corners of the octahedrons, while water molecules, present in the channels and on the surface of aluminum hydroxide fluorides, were hydrogen bonded with the AlF_x_O_6-x_ network. Also, a minor amount of β-AlF_3_ was present in the fresh AF solid [50,51,52].

After calcination of AF in air at 500 °C for 5 h, the resulting sample (AF_c_) showed the XRD spectrum of pure α-AlF_3_ where water and hydroxyl group were completely absent: the corresponding diffraction lines perfectly matched the standard patterns PDF # 44-0231 and 01-080-1007, and the structure ICSD 68826 (See Supplementary Materials for further details. Figures S9–S10). This outcome differed from literature data, reporting instead that β-AlF_3_ was formed by the calcination of AlF_3_·3H_2_O. [47]. Such a discrepancy was not clearly rationalized, but a role was possibly played by the fact that the starting AF sample was a ternary mixture rather than a pure compound. 

The structure of the used catalysts (AF_1_ and AF_2_) was remarkably affected by the nature of the reagents with which these systems came into contact. In particular, if NaCl was absent in the reactant glycerol (**Glyc 1–4**), the corresponding catalyst (AF_1_) did not undergo any appreciable change of its composition: even after 30 h of time-on-stream, the XRD pattern of AF_1_ showed a composition similar to that of the fresh catalyst {AlF_3_·3H_2_O, Al_2_[(OH)_x_F_(1-x)_]_6_(H_2_O)_y_ and β-AlF_3_ in the relative weight amount of 79%, 16%, and 5%, respectively; see SI for further details}. Conversely, if NaCl was present in the glycerol stream, a substantial structural modification of the catalyst took place: after the acetalization of **Glyc5** and **Glyc6** with acetone, the XRD analysis proved that the residual AF_2_ sample was a binary mixture composed of the above described aluminum hydroxide fluoride solid solution {Al_2_[F_1-x_(OH)_x_]_6_(H_2_O)_y_} and a new compound of formula Na_5_Al_3_F_14_, the latter being identified as a Chiolite phase (PDF # 30-1144). These two components were in the relative weight amount of 63.4% and 36.6%, respectively (Figure 4; Rietveld analysis was deposited in the Supplementary Materials section, Figure S8). Of note, with respect to the original AF solid, both AlF_3_·3H_2_O and β-AlF_3_ phases completely disappeared in the used AF_2_ catalyst. 

## 3. Discussion

The here described investigation offers an approach for a rationale design of the catalytic acetalization of glycerol with ketones. The study proves that the choice of the catalyst determines the outcome of the reaction depending on the grade of the reactant glycerol, while the implementation of the process in the continuous flow mode allows to tune T, p, reactant flow rate, and WHSV in order to improve the reaction productivity and to facilitate product isolation.

### 3.1. The Catalyst

Experiments leave few doubts that the reaction of pure or wet glycerol is most efficiently catalyzed by Amberlyst 36 with respect to AlF_3_·3H_2_O: as shown by the model acetalization of **Glyc3** with acetone, at 10 bar, AF requires temperatures as high as 100 °C to offer results comparable to those achieved by using A36 at only 25 °C (Figure 1A and Figure 2A). This different catalytic performance is plausibly due to the effect of strong Brønsted acidity of the resin [53] compared to the weaker acidity of Lewis acid sites and hydroxyl groups on the hydrated aluminum fluoride (see also later on this section) [54,55].

Notwithstanding the thermodynamic limitation that water (as an acetalization by-product) may involve on the equilibrium of the reaction, the activity of both catalysts is only partly affected by the use of wet reagents: under conditions optimized for A36 and AF, steady and good conversions (>80%) are obtained for different types of glycerol containing amounts of water variable in a broad range, from 4 to 25 wt %, respectively (**Glyc3** and **Glyc4**: Figure 1A and Figure 2B). An explanation for this result is offered by the dynamic transfer of reagents and products in the continuous-flow mode that helps the desorption of water from the catalytic bed. In the case of AF, the hydrolytic stability of the catalyst is further proved by the very low leaching of both aluminum and fluoride: the concentration of F^−^ measured in acetal mixtures collected at the reactor outlet is <2 ppm (less than half the U.S. EPA level of 4.0 mg/L allowed in drinking water [56,57]), though, the comparatively higher release of fluoride ions with respect to Al (200 ppb) is plausibly due to some exchange with hydroxide groups at the catalyst surface.

Also, the addition of MeOH (22–30 wt %) to the reactant glycerol has negligible, if any, effects on the performance of both A36 and AF systems (compare **Glyc1** and **Glyc2**: Figure 1A and Figure 2B).

**Scheme 5:**
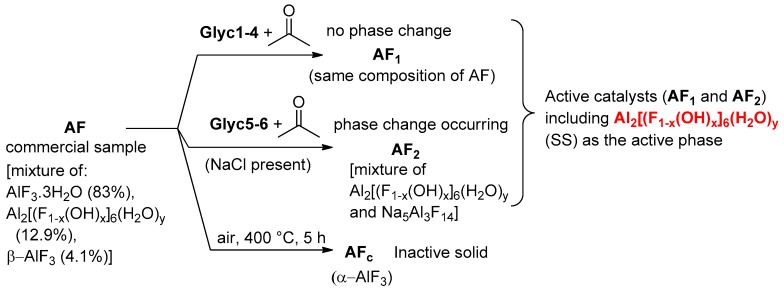
The behavior of commercial AF sample during acetalization tests.

The most intriguing aspect emerging from the comparison of the two catalysts is their different tolerance to the presence of NaCl in the reactant stream. In the reaction of crude-like glycerol (**Glyc5** and **Glyc6**) with acetone, stable conversion and productivity up to 78% and 5.6 h^−1^ are reached by using AlF_3_·3H_2_O as a catalyst (Table 2), while Amberlyst 36 severely deactivates in a few hours, proving it unsuitable for the continuous process where a long catalyst lifetime is imperative (Figure 1B). The exchange of protons with sodium cations (Na^+^) accounts for the progressive decrease of acidity of the organic resin and consequently, for its loss of activity over time. On the other hand, the reasons for the stability of AF to NaCl deserve a more in-depth consideration which may take the cue from the XRD analysis of Figure 3 and Figure 4. A diffractogram of fresh commercial AF proves that the compound is comprised of a mixture of AlF_3_·3H_2_O, a solid solution (SS) of formula Al_2_[(F_1-x_(OH)_x_]_6_(H_2_O)_y_, and very minor amounts of β-AlF_3_ (Figure 3). Such a composition is fully preserved even after a prolonged use (up to 30 h) of AF as a catalyst for the reaction of pure or wet glycerol (**Glyc1–4**) with acetone (Table 4: AF_1_ sample). However, if reactants include NaCl as an additive, then AF undergoes a phase change: of its initial main components, the solid solution {SS: Al_2_[(F_1-x_(OH)_x_]_6_(H_2_O)_y_} remains unaltered, while AlF_3_·3H_2_O is progressively and quantitatively transformed into a chiolite (Na_5_Al_3_F_14_) phase during the CF-acetalization tests. This is clearly proved by XRD spectra of the AF_2_ sample (Figure 4). Although, at the moment, no clear reasons explain why only AlF_3_·3H_2_O is sensitive to a structural modification, the fact that the AF_2_ solid shows a constant catalytic performance for the conversion of crude-like glycerol **Glyc5–6** provides evidence that the SS component is the authentic active phase for the investigated reaction. This is further confirmed by the observation that the calcined AF_c_ compound (pure α-AlF_3_ phase) is no longer an acetalization catalyst. The overall behavior is summarized in Scheme 5.

**Scheme 6:**
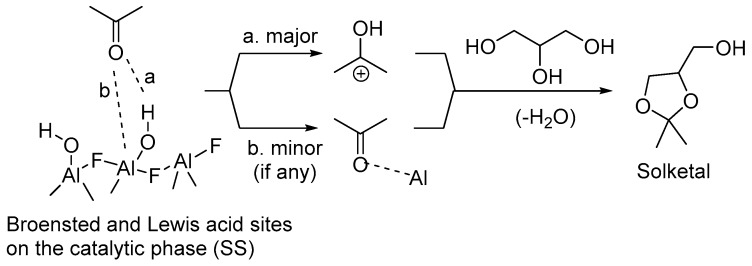
Activation of ketones over Brønsted and Lewis acid sites of the catalyst.

A hypothesis to explain the catalytic role of the SS phase is based on the general mechanism of formation of acetals which starts from the electrophilic activation of the reacting ketones. Accordingly, Scheme 6 shows a pictorial view of the possible interactions (a and b) between the catalyst surface and acetone as a model ketone.

The Brønsted acidity associated to the OH groups of the SS phase offers the major contribution for the initiation of the acetalization reaction (path a, top). While, the lack of activity of anhydrous α-AlF_3_ phase suggests a minor influence, if any, of Lewis acid sites [58] (path b, bottom). In the second step of the reaction, water as a base restores the acidity of the catalyst, and at the same time, it (water) also readily desorbs from the active surface due to the dynamic mass transfer operating in the continuous-flow reactor. The equilibrium position therefore shifts to the right, pushing the reaction forward. Contrarily to the Amberlyst resin, AF keeps on catalyzing the acetalization of crude-like glycerol (**Glyc5–6**) with unchanged conversion and selectivity over time since the (weak) acidity of the hydroxyl groups of the SS phase does not allow exchange reactions with NaCl. Also, a contribution might derive from surface F atoms (of the SS phase) which possibly constitute a hydrophobic shell able to mitigate, if not hinder, the contact of the catalyst with Na cations in the reacting aq. Solution [59]. Whichever the reason, the outcome highlights the potential and the possible synthetic (and economic as well) advantage of AF with respect to the Amberlyst system: since the isolation of acetal products (e.g., solketal) by distillation of final reaction mixtures is not only simpler but also cheaper than techniques required for the purification of off-grade glycerol, [6,26] it is by far more convenient to convert crude glycerol (to acetals) rather than refining the crude reagent and then proceeding with its upgrading.

It should also be noted that the performance of the active SS phase should be evaluated as such rather than in the commercial mixture with AlF_3_·3H_2_O. Although this study is beyond the scope of this paper, future investigations will be focused on the synthesis and applications of the pure SS compound through comparative tests with other acid acetalization catalysts.

### 3.2. The Continuous-Flow (CF) Conditions

CF-conditions may significantly improve the reaction outcome through the optimization of T, p, and reactant flow rate/ratio. This is clear from results of: (i) Figure 1 and Figure 2 which show, for example, how the acetalization of glycerol with acetone can be run at 10 bar well below the previously reported operative pressure (30–120 bar, [27,28,29,30,31]) for the same reaction; (ii) Table 2 and Table 3 which demonstrate how the reaction productivity (P) almost linearly grows by increasing WHSV from 2 to 6 h^−1^. In this range, while the viscosity issue often limits the implementation of CF-processes using glycerol solutions (see Introduction section), nonetheless in our case clogging drawbacks of the reactor have never been experienced. Such an observation probably indicates that a further increase of the reactant flow rate can be applied in a large scale preparation to optimize P, thereby improving the overall performance of the procedure according to the requirement of process intensification. Finally, this also confirms the suitability of AF—even in its powdered commercial form—as a catalyst for the synthesis of GAs.

### 3.3. 2-Butanone

This study proves that the AF catalyst is active not only for the CF-acetalization of crude-like glycerol with acetone, but also with other carbonyl compounds, particularly 2-butanone. Although experimental conditions used for the two ketones are not strictly comparable (see Table 2 and Table 3), 2-butanone appears less reactive than acetone. This trend seems consistent with steric limitations on the kinetics: the more hindered the carbonyl group, the slower its reactions. An analogous behavior has been described also in previous studies on the acetalization of glycerol with different aldehydes, where the process was progressively disfavored by heavier substrates, from butanal to pentanal, hexanal, octanal, and decanal [60].

## 4. Experimental Section

### 4.1. General

Glycerol, acetone, 2-butanone, (2,2-dimethyl-1,3-dioxolan-4-yl)methanol (Solketal) and sodium chloride were ACS grade from Aldrich and were used as received. Aluminum fluoride trihydrate (AlF_3_·3H_2_O, 97%) and Amberlyst 36 (5.4 meq/g, 51%–57% moisture content, 0.2 mL/g total pore volume) were from Aldrich and used as received. Water was of milli-Q grade. GC/MS (EI, 70 eV) analysis were run using a HP5-MS capillary column (L = 30 m, Ø = 0.32 mm, film = 0.25 µm). The following conditions were used. Carrier gas: He; flow rate: 1.2 mL/min; split ratio: 10:1; initial T: 50 °C (3 min), ramp rate: 15 °C/min; final T: 250 °C (3 min). GC/FID analysis were run using an Elite-624 capillary column (L = 30 m, Ø = 0.32 mm, film = 1.8 µm). The following conditions were used. Carrier gas: N_2_; flow rate: 3.5 mL/min; split ratio: 1:10; initial T: 100 °C (0 min), ramp rate: 15 °C/min; final T: 220 °C (5 min). ICP-OES analysis were run using a Perkin Elmer Optima 5300DV (PerkinElmer, Waltham (MA), USA). ^1^H-NMR were recorded at 300 MHz, ^13^C spectra at 75 MHz and chemical shift were reported in δ values downfield from TMS; CDCl_3_ was used as solvent.

XRPD patterns were recorded at room temperature with a step size of 0.05° in the 5°–100° range. The diffraction data were collected (10 s·step^−1^) using a Philips X’Pert system (PW3020 vertical goniometer and PW3710 MPD control unit, PhilipsPANanalytical, Lissone, Italy) equipped with a focusing graphite monochromator on the diffracted beam and with a proportional counter (PW1711/90) with electronic pulse height discrimination. A 0.5° divergence slit was used, together with a receiving slit of 0.2 mm, an antiscatter slit of 0.5° and Ni-filtered Cu *K*α radiation (30 mA, 40 kV).

### 4.2. CF-Apparatus

**Figure 5:**
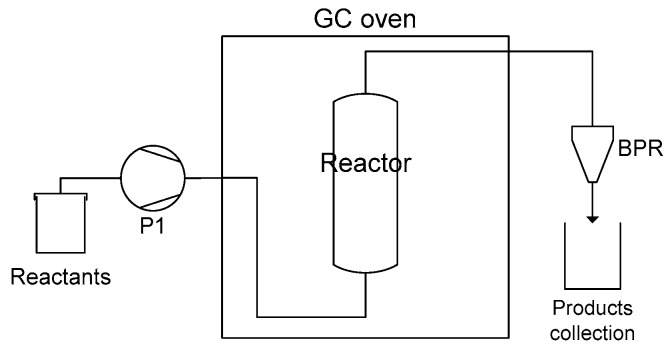
Experimental setup used for the continuous-flow acetalization of glycerol with acetone and 2-butanone.

The apparatus used for the investigation was assembled in-house (Figure 5). A Shimadzu LC-10AS HPLC pump (P1) was used to deliver liquid reactants to stainless steel tubular reactor. Two reactor sizes were considered: (a) L = 12 cm, Ø = 1/4″, inner volume = 0.875 cm^3^; and (b) L = 12 cm, Ø = 3/8″, inner volume = 3.5 cm^3^. Whatever the size, the reactor was filled with the catalyst (Amberlyst 36 or AlF_3_·3H_2_O), placed in the upright position in a gas chromatographic oven (GC oven), and heated at the desired temperature. A JASCO BP-2080 back pressure regulator (BPR), placed at the outlet of the reactor, was used to keep the pressure constant throughout the system.

#### Safety Warning

Operators of high pressure equipment should take proper precautions to minimize the risk of personal injury.

### 4.3. General Procedure for the CF Acetalization Reactions of Glycerol

#### 4.3.1. Preparation of Reactants

Six different types of glycerol were considered for the study (**Glyc1–6**). These were all prepared by mixing ACS-grade glycerol (**Glyc1**, 10 g) with different amounts of water (0.5 to 11 mL), methanol (1.26 to 11.36 mL), and NaCl (0.5 g) in order to reach the compositions described in Table 1.

All the CF-acetalization reactions were carried out according to the following operations:

#### 4.3.2. Reaction Procedure

A homogeneous solution of glycerol (**Glyc1–6**) and the desired ketone (acetone or 2-butanone) in the molar ratio (Q = ketone:glycerol) variable from 4 to 60 was delivered to the above described CF-reactor previously filled with Amberlyst 36 (0.9 g) or with AlF_3_·3H_2_O (depending on the size of the reactor, the amount of catalyst was 0.67 g or 4.2 g, respectively). Each reaction was run at a constant flow mode. However, the explored range of flow rates of the reactants mixture was from 0.1 to 1.18 mL/min. The operating pressure and temperature were set and checked at the desired values (10–35 bar, and 25–100 °C, respectively) by the use of BPR and the GC oven described in Figure 5. The apparatus was preliminarily conditioned for 1 h. Afterwards, the reaction mixture was sampled at time intervals of 60 min, for a minimum of three samples, and analyzed by GC/FID and GC/MS.

#### 4.3.3. System Cleaning and Reuse of the Reactor

Once the experiment was completed, if the catalytic bed was reused, the GC oven was set to 50 °C and pure acetone (50 mL at 0.5 mL/min) was delivered to the reactor. In the specific case where the reactants mixture contained sodium chloride, Milli-Q water was delivered to the reactor (50 mL at 0.5 mL/min) prior to the acetone cleaning. Afterwards, the pump was stopped, the system was vented to the atmospheric pressure, and the GC oven was allowed to cool at rt.

### 4.4. Aluminium and Fluoride Contents by ICP-OES and Ionic Chromatographic Analyses

In the presence of AF as a catalyst, the acetalization of **Glyc3** and **Glyc4** with acetone was carried out according to the above described procedure (100 °C, 10 bar: conditions of Figure 2B). Once experiments were complete, the mixtures sampled at the reactor outlet were used as such ionic chromatographic analyses or they were rotary evaporated (60 °C, 40 mbar, 1 h), and the oily residues were subjected to ICP analyses (details are reported in the SI section).

Under the same conditions, an additional experiment was carried out as a blank test in the absence of AlF_3_·3H_2_O. The residue achieved after the test was also analyzed by ICP and ionic chromatography.

### 4.5. Isolation and Characterization of Products

#### 4.5.1. (2,2-dimethyl-1,3-dioxolan-4-yl)methanol (Solketal)

The product was isolated from two reactions (A and B) starting from **Glyc1** and **Glyc5**, respectively.

The acetalization of Glyc1 with acetone (Q = 40) carried out under the conditions of Figure 2B (100 °C and 10 bar; flow rate: 0.2 mL/min; AlF_3_·3H_2_O: 0.67 g). The reaction was allowed to proceed for 15 h. The homogeneous mixture recovered at the reactor outlet was rotary evaporated (60 °C, 40 mbar, 1 h) and distilled (70 °C, 40 mbar). The title product was obtained as a colorless mixture of isomers **1b** and **1b′** (ratio **1b**/**1b′**~50) in a 74% overall yield (5.8 g, purity 98% by GC/FID).

The acetalization of **Glyc5** with acetone (Q = 4) was carried out under the conditions of Figure 2B (100 °C and 10 bar; flow rate was: 0.34 mL/min; AlF_3_·3H_2_O: 0.67 g). The reaction was allowed to proceed for 3 h. The homogeneous (pale green) mixture recovered at the reactor outlet was rotary evaporated (60 °C, 40 mbar, 1 h), filtered to remove the solid residue of sodium chloride, and distilled (70 °C, 40 mbar). The title product was obtained as a colorless mixture of isomers **1b** and **1b′** (ratio **1b**/**1b′**~50) in a 59% overall yield (11.5 g, purity 98% by GC/FID). The structure of the product was confirmed by ^1^H-NMR, ^13^C-NMR and GC/MS and by comparison to an authentic commercial sample of solketal.

#### 4.5.2. (2-Ethyl-2-methyl-1,3-dioxolan-4-yl)methanol (**1c**)

The product was isolated from the acetalization of **Glyc1** with 2-butanone (Q = 60) carried out under the conditions of entry 1 in Table 3 (100 °C and 10 bar; flow rate: 0.2 mL/min; AlF_3_·3H_2_O: 0.67 g). The reaction was allowed to proceed for 24 h. The homogeneous mixture recovered at the reactor outlet was rotary evaporated (60 °C, 40 mbar, 1 h) and distilled (79 °C, 40 mbar). The title product was obtained as a colorless mixture in a 68% overall yield (5.2 g, purity 96% by GC/FID). It was characterized by ^1^H-NMR, ^13^C-NMR, and GC/MS.

^1^H-NMR mostly shows multiplets due to a partial overlap of the signals of *cis*- and *trans*-isomers of **1c** present in approximately equal concentrations.

^1^H-NMR (300 MHz, CDCl_3_) δ (ppm): 4.25 (m, 2H), 4.12–4.00 (m, 2H), 3.85–3.72 (m, 4H), 3.61 (m, 2H), 1.79–1.62 (m, 4H), 1.39 (s, 3H), 1.33 (s, 3H), 0.96 (m, 6H). 13C-NMR (75 MHz, CDCl_3_) δ (ppm): 111.91, 111.61, 76.93, 76.25, 66.23, 66.19, 63.46, 63.28, 32.90, 31.99, 24.54, 23.45, 8.86, 8.57. GC/MS (relative intensity, 70 eV) *m*/*z*: 145 (M^+^, <1%), 131 (20), 117 (100), 115 (27), 86 (11), 73 (11), 61 (14), 57 (84), 55 (18), 43 (80).

Further characterization details are available in the Supplementary Materials section (Figures S1–S5).

## 5. Conclusions

Catalysts for the implementation of robust CF-methods for organic synthesis must be active, durable (long-lived compounds), and versatile so as to accommodate reactant feeds with variable chemical compositions. In the specific case of the CF-acetalization of glycerol with ketones, this study demonstrates that the two investigated catalysts, Amberlyst 36 and AlF_3_·3H_2_O, respectively, possess complementary activities and features. The first one (A36) is the most efficient system: it is capable of operating under very mild conditions, but it is readily poisoned by even small amount of NaCl which is a common contaminant of crude glycerol. The second compound (AF) requires a higher operating temperature, but its performance is insensitive to the presence of inorganic salts, being thereby suitable for the conversion of different kinds of raw glycerol. Overall, the approach reported here shows that the design of a continuous-flow synthesis of glycerol acetals can be conveniently tailored according to the quality of the starting materials, by using either conventional acid organic resins or introducing new acetalization catalysts such as AF. Both catalysts, when used at optimized T, p, and flow rates (WHSV) afford stable activity in the long run, though AF may offer a more attractive standpoint for the straightforward valorization of crude glycerol.

Finally, this study provides evidence that the activity of the investigated commercial sample of AF is due to the presence of a solid solution (SS) composed of an aluminum hydroxide fluoride phase {Al_2_[F_1-x_(OH)_x_]_6_(H_2_O)_y_}. As a future perspective, the potential of this result will be examined by synthesising the SS phase in a pure form and testing it as such for both acetalizations and other acid catalyzed model reactions under CF-conditions.

## Figures and Tables

**Table 1:** Different types of glycerol used in this work.

Entry	Glycerol: Additives, Molar Ratio ^b^ (wt %, Composition)				Reactant Label
	Glycerol ^a^	MeOH	H_2_O	NaCl	
1	(100)	none	none	none	**Glyc1**
2	1 (70)	1.2 (30)	none	none	**Glyc2** ^c^
3	1 (68)	1.2 (28)	0.3 (4)	none	**Glyc3** ^c^
4	1 (53)	1.2 (22)	2.4 (25)	none	**Glyc4** ^c^
5	1 (49.5)	1.2(22)	2.4 (24)	0.08(2.5)	**Glyc5** ^c^
6	1 (44.3)	0.3 (4.4)	5.6 (49)	0.08 (2.3)	**Glyc6** ^c^

^a^ ACS grade glycerol was used in all experiments; ^b^ Glycerol:additive molar ratio. The % wt composition of the reactant glycerol is shown in parenthesis; ^c^ Relative proportions of MeOH, water, and NaCl were adjusted based on references [16,23].

**Table 2:** Comparison of acetalization of **Glyc5** and **Glyc6** with acetone catalyzed by AlF_3_·3H_2_O ^a^.

Entry	Reactant	AlF_3_·3H_2_O (g)	Q Ratio (mol:mol) ^b^	WHSV	Conv.’n (%) ^c^	Productivity (P) (g of (1b + 1b′)/g_cat_·h)
1	**Glyc5**	0.67	4	2	78	2.2
2			4	4	67	3.8
3			4	6	65	5.6
4		4.2	4	2	75	2.2
5	**Glyc6**	0.67	4	2	30	0.86
6			8	2	60	1.7
7			8	4	71	4.0
8		4.2	8	2	54	1.6

^a^ All reactions were carried out at 100 °C and 10 bar; ^b^ Acetone:glycerol molar ratio; ^c^ Conversion of glycerol determined after 24 h.

**Table 3:** The acetalization of **Glyc1** and **Glyc5** with 2-butanone catalyzed by AlF_3_·3H_2_O ^a^.

Entry	Reactant	Q Ratio (mol:mol)	WSHV	Conv.’n (%) ^b^	Productivity (P) (g of (1c + 1c′)/g_cat_ h)
1	**Glyc1**	60	2	85	2.7
2	**Glyc6**	4	2	45	1.3

^a^ All reactions were carried out at 100 °C and 10 bar; ^b^ Conversion of glycerol after 24 h. The reported value did not differ from that measured after the first two hours of reaction.

**Table 4:** Different samples of fresh, calcined, and used AlF_3_·3H_2_O considered for characterization analyses.

Entry	Sample	Label	Time-on-Stream (h) ^a^
1	AlF_3_·3H_2_O Fresh	AF	-
2	Calcined AlF_3_·3H_2_O ^b^	AF_c_	-
3	AlF_3_·3H_2_O after use for **Glyc1-4**	AF_1_	30
4	AlF_3_·3H_2_O after use for **Glyc5-6**	AF_2_	90

^a^ Total time of use of the catalyst in the acetalization of **Glyc1–4** and **Glyc5–6** with acetone: entry 3 and 4, respectively. Reaction conditions were described in Figure 2 and Table 2; ^b^ AF_c_ was obtained by calcination of AF in air at 500 °C and 5 h.

## Supplementary Materials

Supplementary materials can be accessed at: http://www.mdpi.com/1420-3049/21/5/657/s1.
