# Supplementary material for: Towards a Rational Design of a Continuous-Flow Method for the Acetalization of Crude Glycerol: Scope and Limitations of Commercial Amberlyst 36 and AlF3·3H2O as Model Catalysts
Source: Molecules. 2016 May 18;21(5):657. doi: 10.3390/molecules21050657 (PMC6274036; doi:10.3390/molecules21050657)
Supplement: Supplementary file 1 [file molecules-21-00657-s001.pdf]

Supplementary Materials: Towards a Rationale Design of a Continuous-Flow Method for the Acetalization of Crude Glycerol: Scope and Limitations of Commercial Amberlyst 36 and $AlF_3·3H_2O$ as Model Catalysts

Sandro Guidi, Marco Noè, Pietro Riello, Alvise Perosa and Maurizio Selva

ICP Analysis

ICP-OES analyses were carried out to evaluate the leaching of Al from the catalytic bed of commercial $AlF_3·3H_2O$ used in this investigation. Analyses were run on a Perkin Elmer Optima 5300 DV in axial direction at 394.401 nm. A calibration curve was obtained by using seven aqueous solutions containing 300, 200, 150, 100, 60, 40 and 20 ppb of Al. These solutions were all prepared by dilution of a 1000 mg/L standard solution of ionic Al in HNO_3. The linear fit was automatically calculated by the ICP software resulting with interceptor = −151.8, slope = 21.70 and correlation coefficient = 0.996961.

A total of three samples were considered for Al-analyses. They were obtained according to the procedure described in the experimental section. The first two samples (A and B) derived from the reaction of **Glyc3** and **Glyc4** (see Table 1, main text) with acetone catalyzed by $AlF_3·3H_2O$. While, the third one was prepared by flowing the reactants glycerol (**Glyc3**) and acetone through the CF-apparatus in the absence of the catalyst (Blank).

Before any measure, each sample was first diluted with milli-Q water (20 mL). A and B were then diluted again in a 1:3 *v*/*v* ratio. Table S1 reports the results. Each analysis was the average of 6 subsequent acquisitions.

Table S1. ICP-OES analyses of the Al content.

Entry	Sample	Reactant	Al Content (μg/L)
1	A	**Glyc3**	214.0
2	B	**Glyc4**	188.1
3	Blank	**Glyc3**	137.4

Ion Chromatography Analysis

Ion chromatography analyses were carried out to evaluate the leaching of F^- from the catalytic bed of $AlF_3·3H_2O$ used in this investigation. Analyses were run on a Dionex LC20 (Chromatography enclosure) equipped with a Dionex GP40 gradient pump and a Dionex ECD ED40 (working at 100 mA). A Dionex AS14 was used as column with 1mM carbonate/3.5 mM bicarbonate as a mobile phase. A calibration curve was obtained by using four aqueous solutions containing 0.5, 1, 3, 7 ppm of F^-. The linear fit was automatically calculated by the chromatograph control software (Chromeleon) resulting with slope = 0.131, interceptor forced to 0 and correlation coefficient = 0.999868.

A total of two samples were considered for F^- analyses. They were obtained according to the procedure described in the experimental section. The first sample (A) was derived from the reaction of glycerol (**Glyc3**) with acetone catalyzed by $AlF_3·3H_2O$ while, the second one was prepared by flowing the reactants in the absence of the catalyst (blank).

Before any measure, each sample was first diluted with milli-Q water in a 1:5 *v*/*v* ratio. Table S1 reports the results. Each analysis was the average of 4 subsequent acquisitions.

Table S2. Ion chromatography analyses of the F^- content.

Entry	Sample	F^- Content (mg/L)
1	A	2.808
2	Blank	0.274

^{1}H NMR Spectrum of 1c (1:1 Mixture of cis and Trans Isomers)

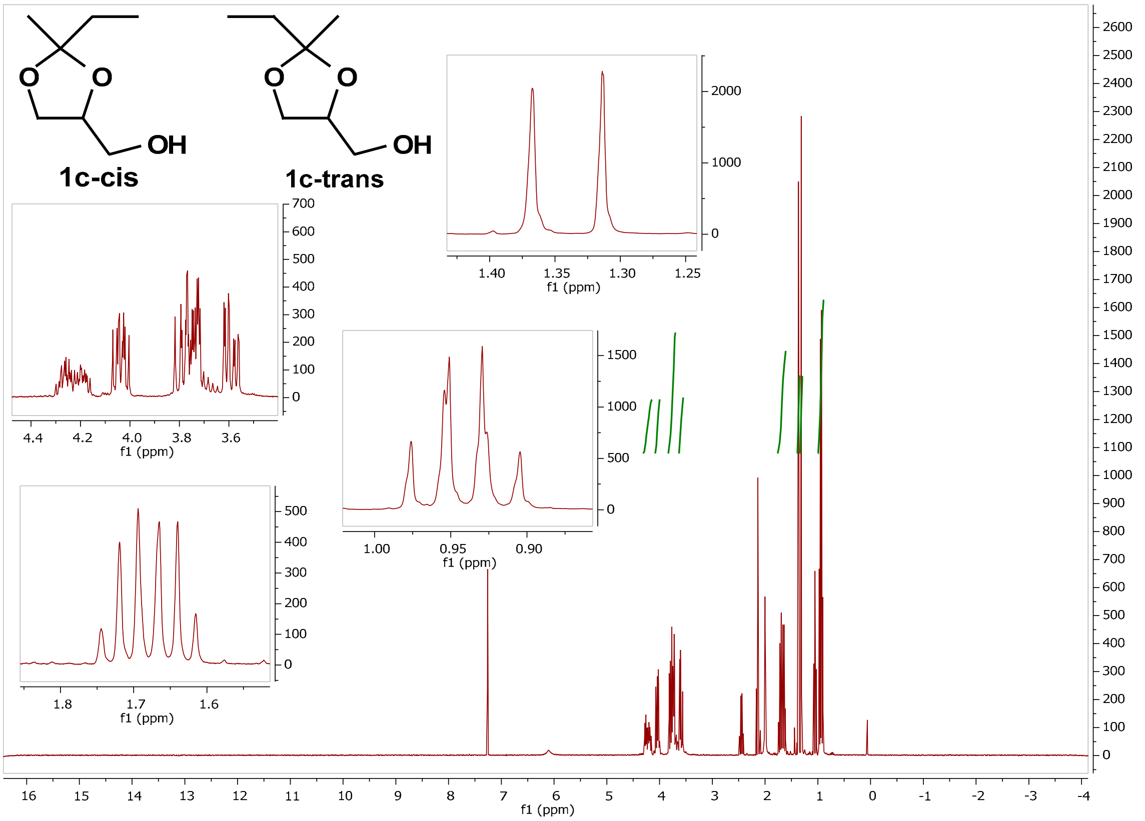

Figure S1. ^{1}H NMR spectrum of 1c.

^{1}H-NMR (300 MHz, $CDCl_3$) δ (ppm): 4.25 (m, 2H), 4.12–4.00 (m, 2H), 3.85–3.72 (m, 4H), 3.61 (m, 2H), 1.79–1.62 (m, 4H), 1.39 (s, 3H), 1.33 (s, 3H), 0.96 (m, 6H).

The spectrum also showed traces of 2-butanone which corresponded to the following signals: [1] ^{1}H-NMR (300 MHz, $CDCl_3$) δ (ppm): 2.47 (q, *J* = 7.3 Hz, 2H), 2.15 (s, 3H), 1.07 (t, *J* = 7.3 Hz, 3H).

^{13}C NMR Spectrum of 1c (1:1 Mixture of cis and Trans Isomers)

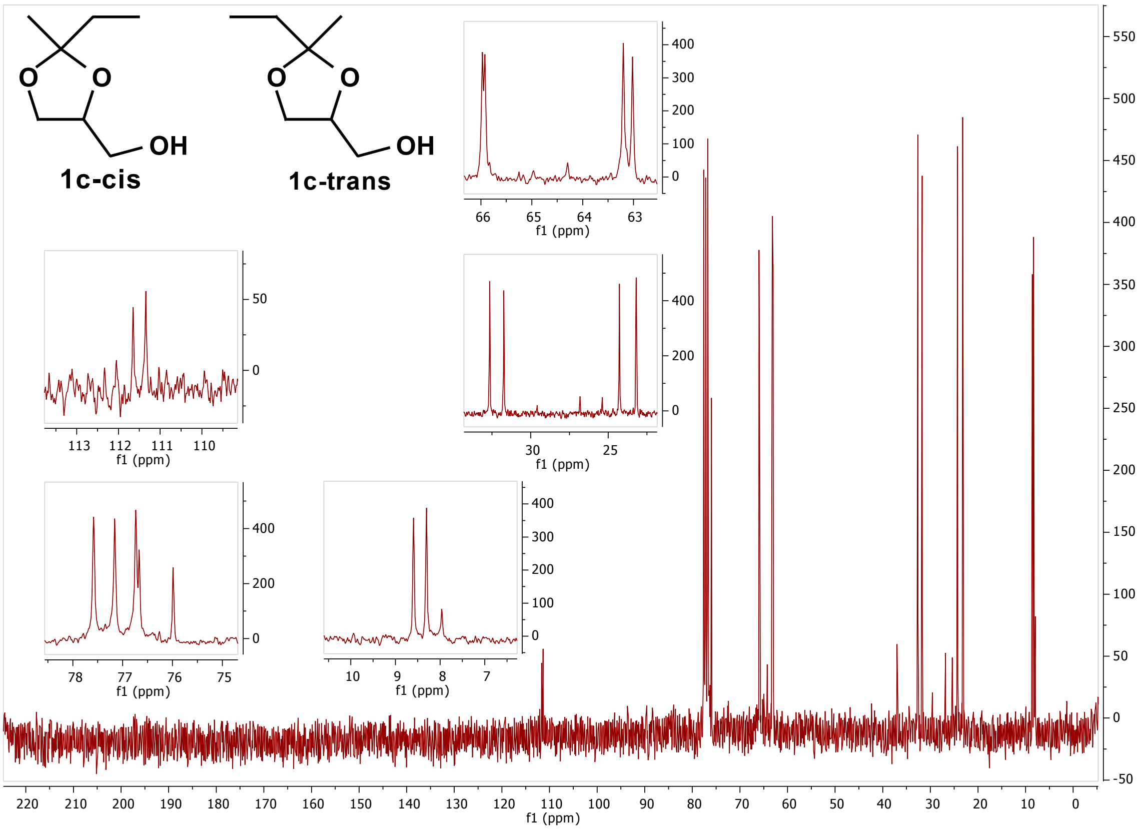

Figure S2. ^{13}C NMR spectrum of **1c**.

^{13}C-NMR (75 MHz, $CDCl_3$) δ (ppm): 111.91, 111.61, 76.93, 76.25, 66.23, 66.19, 63.49, 63.28, 32.90, 31.99, 24.54, 23.45, 8.86, 8.57.

The spectrum also showed traces of 2-butanone which corresponded to the following signals: [1] ^{13}C-NMR (75 MHz, $CDCl_3$) δ (ppm): 37.00, 29.59, 7.96.

MS Spectrum of 1c (1:1 Mixture of cis and Trans Isomers)

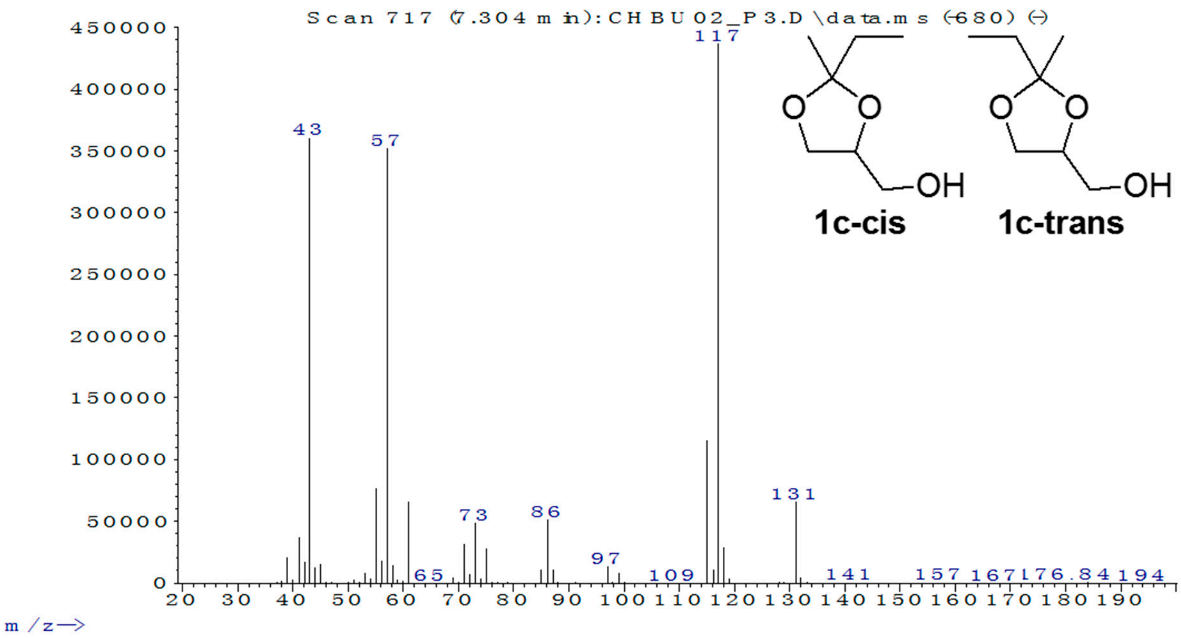

Figure S3. MS spectrum of **1c**.

GC/MS (relative intensity, 70eV) m/z: 146 (M^+, <1%), 131 (20), 117 (100), 115 (27), 86 (11), 73 (11), 61 (14), 57 (84), 55 (18), 43 (80).

MS Spectrum of 1c′

Figures S4 and S5 report MS spectra of the two minor products observed in the reaction of glycerol with 2-butanone. Signals are consistent with the structure of the following cyclic six-membered ring acetals:

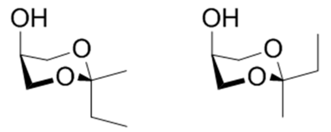

1c'-trans **1c'-cis**

However, it is not possible to establish which isomer corresponds to which MS spectrum

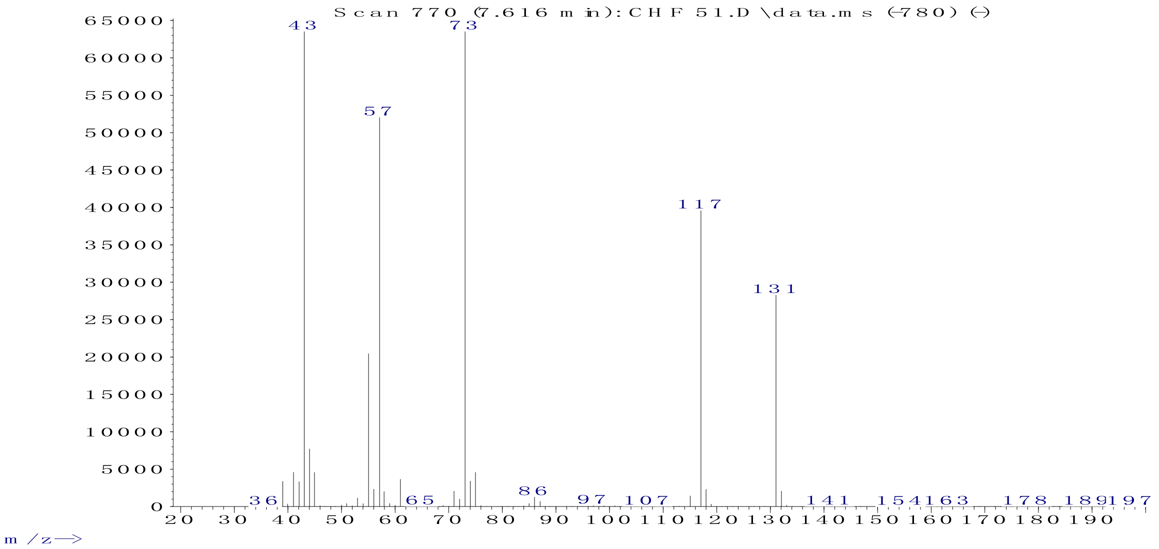

Figure S4. MS spectrum of **1c′** (cis or trans).

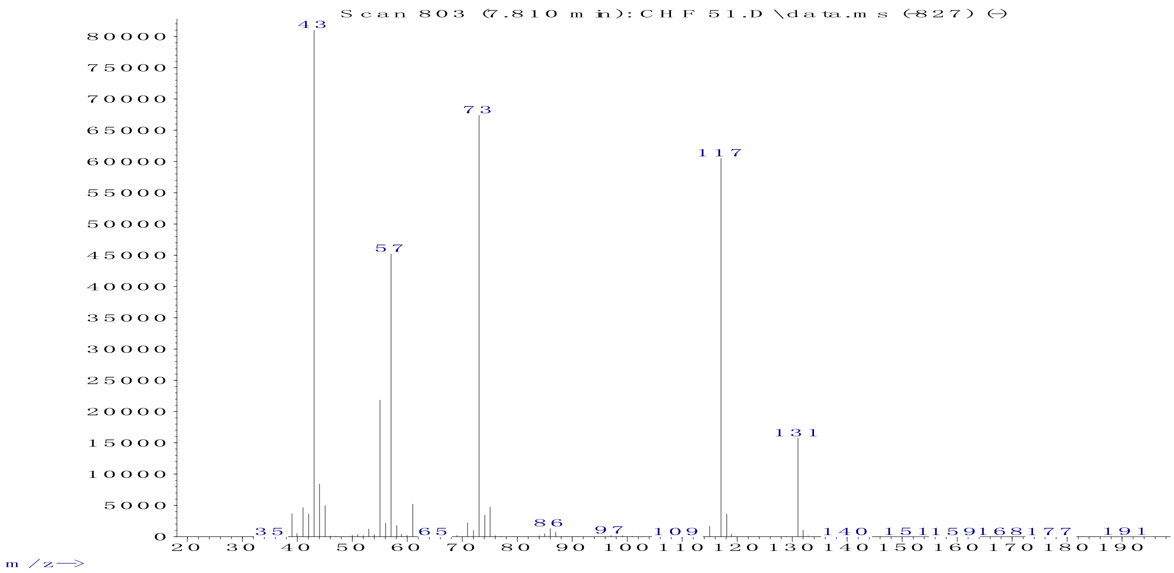

Figure S5. MS spectrum of **1c′** (cis or trans).

GC/MS (relative intensity, 70eV) m/z:

Figure S4: 146 (M^+, <1%), 131 (43), 117 (61), 73 (99), 57 (80), 55 (31), 44 (12), 43 (100).

Figure S5: 146 (M^+, <1%), 131 (22), 117 (82), 73 (85), 57 (56), 55 (27), 44 (10), 43 (100).

XRD Analysis

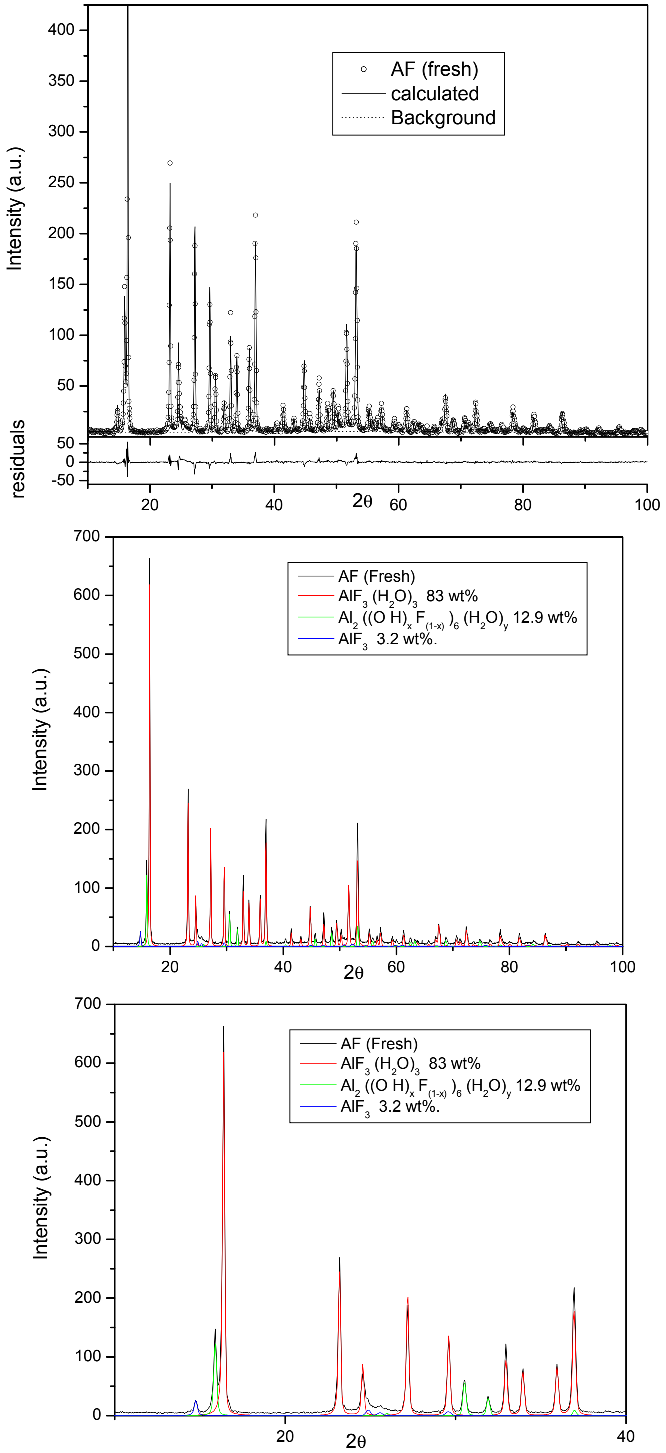

Figure S1. XRD pattern of commercial $AlF_3 \cdot 3H_2O$ (Fresh, black pattern) compared with three reported different AlF_3 phases (red, green and blue patterns).

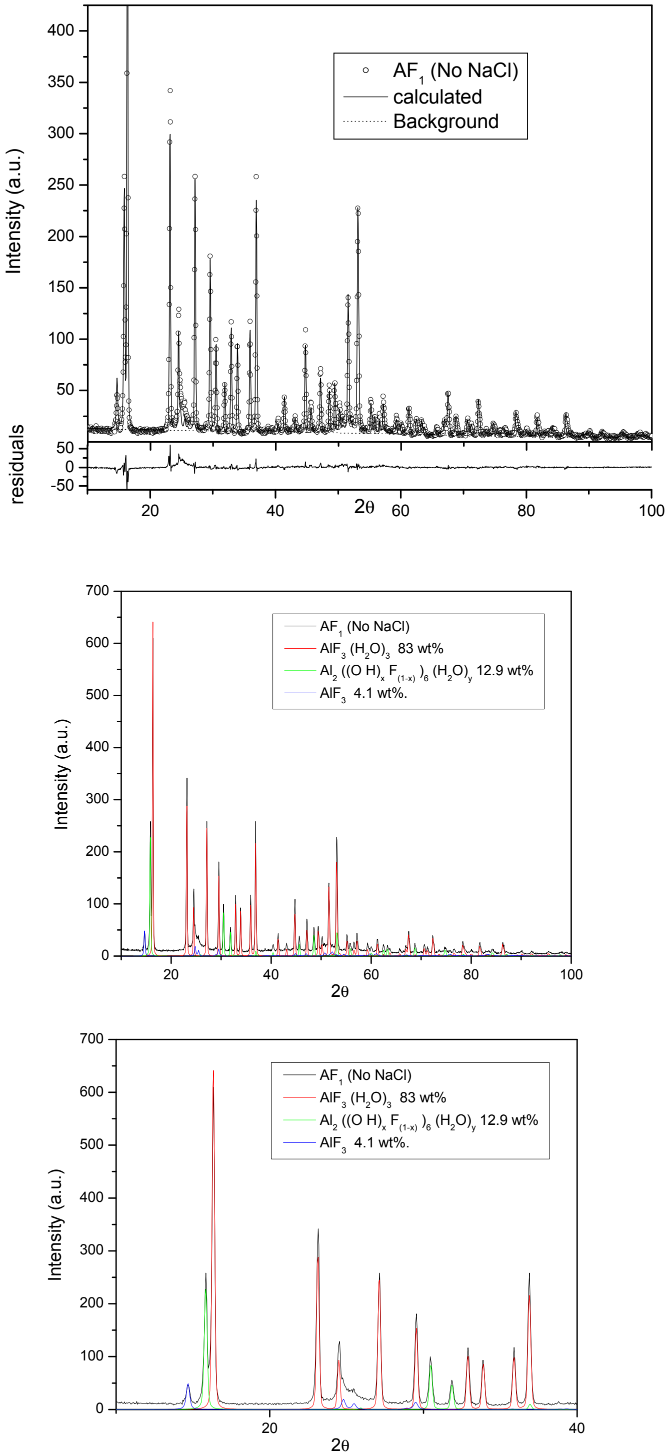

Figure S2. XRD pattern of $AlF_3{\cdot}3H_2O$ used without sodium chloride (No NaCl, black profile) compared with XRD profiles of the three different phases present in the fresh AF sample (red, green and blue profiles).

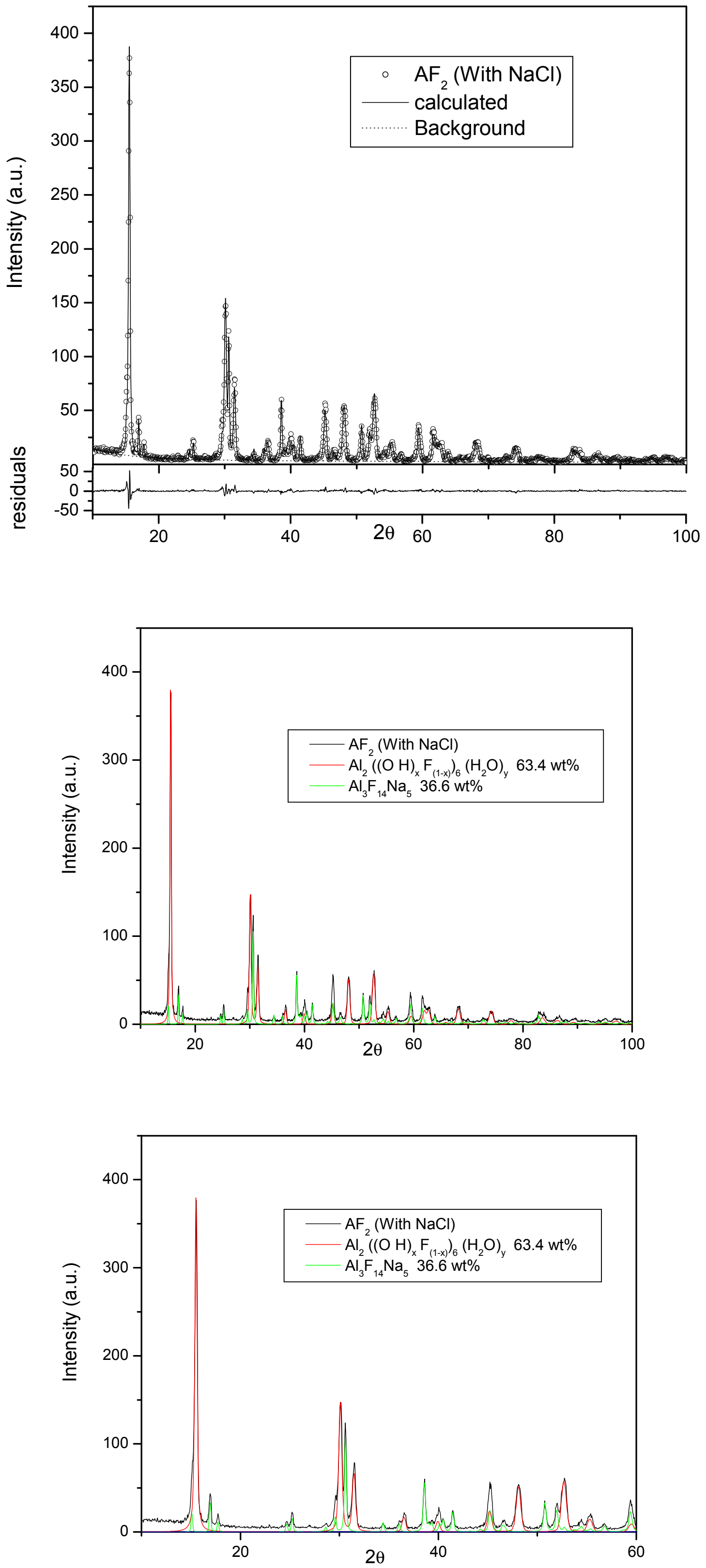

Figure S3. XRD pattern of $AlF_3{\cdot}3H_2O$ used with sodium chloride (With NaCl, black profile) compared with XRD profiles of $Al_2[(F_{1-x}(OH)_x]_6(H_2O)_y$ and Chiolite ($Na_5Al_3F_{14}$) phases (red and green profiles).

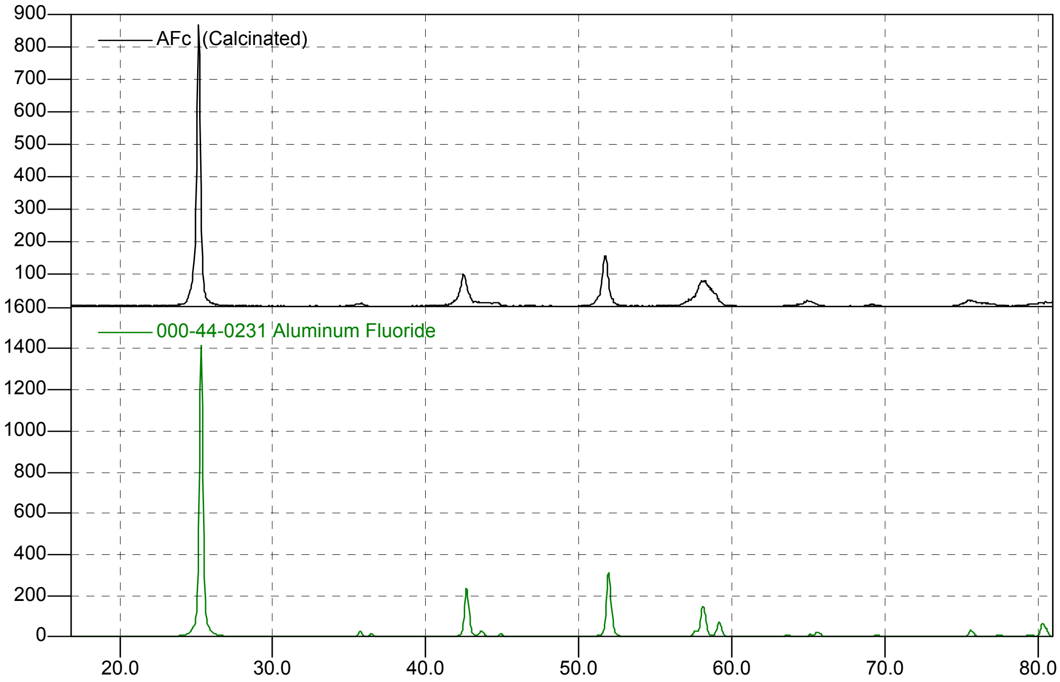

Figure S4. XRD pattern of calcined $AlF_3{\cdot}3H_2O$ in air at 500 °C for 5 h (black) compared with the reported pattern of α-$AlF_3{\cdot}3H_2O$ (green).

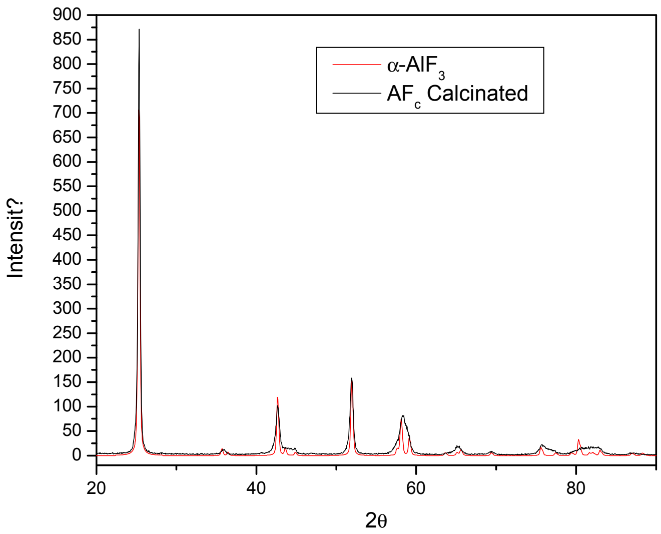

Figure S5. XRD pattern of commercial calcined $AlF_3{\cdot}3H_2O$ in air at 500 °C for 5 h (black) compared with the reported α-AlF_3 pattern (red).

References

1 Gottlieb, H.E.; Kotlyar, V.; Nudelman, A., NMR Chemical Shifts of Common Laboratories as Trace Impurities. *J. Org. Chem.* **1997**, *62*, 7512–7515.
